# The intersection of digital health and artificial intelligence: Clearing the cloud of uncertainty

**DOI:** 10.1177/20552076251315621

**Published:** 2025-01-24

**Authors:** Pooyeh Graili, Bijan Farhoudi

**Affiliations:** 1 661942 Quality HTA, Oakville, Ontario, Canada; 2 Department of Master of Health Informatics, Institute for Health Policy, Management and Evaluation, 274071 Dalla Lana School of Public Health, 7938 University of Toronto, Toronto, Ontario, Canada; 3 Information Technology Management, 120481 Ted Roger School of Management, Toronto Metropolitan University, Toronto, Ontario, Canada; 4 3146 Independent Researcher, Calgary, Alberta, Canada

**Keywords:** Digital health (DH), digital health technologies (DHTs), digital health services (DHSs), artificial intelligence, real-world data (RWD), machine learning (ML)

## Abstract

Digital health (DH) and artificial intelligence (AI) in healthcare are rapidly evolving but were addressed synonymously by many healthcare authorities and practitioners. A deep understanding and clarification of these concepts are fundamental and a prerequisite for developing robust frameworks and practical guidelines to ensure the safety, efficacy, and effectiveness of DH solutions and AI-embedded technologies. Categorizing DH into technologies (DHTs) and services (DHSs) enables regulatory, HTA, and reimbursement bodies to develop category-specific frameworks and guidelines for evaluating these solutions effectively. DH is the key in generating real-world data, which is increasingly important in decision-making processes. The potential benefits of DHTs in improving health outcomes and reducing health system costs can position them alongside traditional health technologies in certain medical conditions. AI, one of the potential tools for DH, can be embedded in technologies, such as medical devices or applications, to enhance functionality and performance. AI excels at handling numerical and perceptual data. In the context of numerical data, machine learning algorithms enable prediction, classification, and clustering. In managing perceptual data, AI recognizes image/video, voice, and text. In recent years, generative AI, a form of AI that generates new content by employing a combination of a wide range of learning approaches, has become prominent in research and influences the health sector. A thorough understanding of DH and AI, along with accurate terminology use, would facilitate the timely generation of regulatory and HTA-grade evidence that helps improve health outcomes and decision-making certainty.

## Introduction

Digital health (DH) and artificial intelligence (AI) solutions are exponentially emerging and claim to impact health and time. Whereas the developers of these solutions fully understand the technical fundamentals, they may not be well-versed in established health sector principles, regulations, and standard clinical practices. This results in creating solutions that have not been thoroughly analyzed for their impacts on patients or health systems.

Health systems must enhance efficiency, expedite decision-making, and provide access to health technologies and services while ensuring safety, avoiding potential harm(s), and promoting the effectiveness of any solution to human physical and mental health before approving new health technologies. To minimize the risks of the costly and sometimes irreversible impact of DH and AI, healthcare experts and decision-makers must first be clear about these concepts. Such clarity is essential for developing robust frameworks, establishing quality standards and thresholds, adapting evaluation methods, addressing ethical implications, and developing validation and harmonization methodologies. While regulatory and HTA bodies are central to establishing comprehensive frameworks for the ethical and effective deployment of DH and AI solutions, input from healthcare professionals is vital to align these frameworks and remain grounded in real-world clinical needs. Their expertise contributes to aligning macro-level guidelines with patient-centred care principles, thus bridging the gap between regulatory goals and practical application. Furthermore, it enables the formulation of practical guidelines and standard frameworks with explicit information on evidence requirements on technologies and the impact of emerging technologies and services on health.^1–4^

## Digital health

As a broad term, DH leverages digital platforms and tools to promote well-being, manage health conditions, and improve healthcare delivery systems. DH solutions can help with prevention, treatment, diagnosis, rehabilitation, and long-term care. DH refers to digital health technologies (DHTs) and digital health services (DHSs), but they are not distinct. While several organizations offer somewhat similar definitions of DH, the lack of complete coverage of the same categories leads to the definition unclarity.^5–8^ Consequently, it becomes challenging to develop overarching guidelines and frameworks that encompass both categories. Given the disparity in the nature of these two categories, a clearer definition of DH technologies and services necessitates the development of category-specific guidelines and frameworks for defining data quality standards, harmonizing measured outcomes, evaluation and validation methods. This simplification allows regulatory, HTA, and reimbursement bodies to clarify their requirements and further enhance their processes to adopt new DH solutions.

One category of DH solutions, DHTs, includes but is not limited to mHealth, wearables, environmental sensors, smartphones and tablets, robotics, software, and AI-embedded medical devices. The other group of DH solutions, like electronic medical records (EMR) and electronic health records (EHR), telehealth/telemedicine, and virtual care, can be categorized as DHSs. Both solutions offer potential benefits for improving patients’ health outcomes and the efficiency of health care by increasing the systems’ capacity while reducing costs.

Beyond these potential benefits, DH solutions generate substantial real-world data (RWD) collected by healthcare professionals and patients. The real-world evidence derived from this data can be considered reliable sources for decision-making when following established criteria and frameworks for high-quality data generation. It is especially significant for regulatory and HTA purposes, as there is a growing interest in leveraging complementary sources of evidence to make well-informed decisions with greater certainty and/or continue reimbursement for the real value for money.^9,10^

DHTs also have the potential to offer a range of opportunities, such as enhancing the effectiveness of conventional health technologies. A great example can be found in the applications used to improve the management of hyperglycemia in diabetic patients. These applications help patients manage their medications, boost adherence rates, and improve their outcomes. Other medical applications may compete with conventional health technologies like those developed for treating depression within specific severity levels. When such an application demonstrates equal or greater effectiveness than medications, it may serve as a medication substitute, for example, in mild to moderate depression.^11^ The advantage lies in offering a new treatment modality while avoiding chemical side effects and potential interactions with other medications, especially for patients with multiple co-morbidities. Such an option can also be promising for older patients who take several medications daily. DHTs can be employed to improve sleep cycles, exercise more, eat healthy, and other public health interventions that lead to healthy lifestyles, prevent non-communicable diseases and save huge costs for the health systems. These digital technologies can be categorized as digital tech for all (DTA) and digital tech for professionals (DTP), which require prescriptions, or their generated data can be relied on for technology assessment.

Integrating DHTs also enables continuous, real-time, and remote data collection opportunities directly from patients during clinical trials and post-market surveillance.^1,10^ This may significantly reduce R&D costs and potentially lower drug prices. Therefore, reimbursement systems may require new payment considerations to optimize investment and market uptake.

Given these contexts, it is essential to categorize and prioritize DHTs based on their impact on the collected RWD and measured endpoints. The impact on patients’ outcomes is particularly crucial in the regulatory, HTA, and reimbursement processes. Data reliability and security, and patient privacy must be carefully managed when using DHT for evidence generation; however, transparent and robust frameworks and category-specific guidelines are prerequisites.^9^

## Artificial intelligence

AI, one of the tools potentially used to develop DH solutions, is a discipline within computer science. It focuses on creating systems capable of performing tasks that typically require human intelligence and cognitive function. These tasks encompass complex activities such as reasoning, problem-solving, perceiving and learning, all achieved through computational models and algorithms. Anticipated advancements in AI accuracy, if aligned with healthcare standards, can significantly augment its role in the health sector over the next decade. While AI tools offer numerous benefits, they also raise ethical concerns. Principles such as transparency, patient privacy and autonomy, data security, equity, fairness, and beneficence must be addressed in advance to ensure the responsible use of AI in healthcare. Explicit guidelines and ethical frameworks are essential for developing, deploying, and evaluating AI-based solutions, ensuring they align with these principles and promote trust and accountability in healthcare applications. AI can be embedded in health technologies, such as medical devices, wearables, mobile applications, or web platforms, to enhance their functionality and performance or to leverage collected data to advance precision and personalized medicine.^4,12–18^ AI tools have the potential to reduce human errors because of their ability to decrease human workload and empower individuals to think and perform at a higher level of quality. It is essential to acknowledge that neither human nor AI tools are 100% accurate in the health system. Still, their combination makes achieving optimal outcomes for patients and societies possible.

AI excels in handling numerical and perceptual data and covers diverse domains, such as machine learning (ML), natural language processing (NLP), rule-based systems, robotics, and more.^12,13^ In recent years, generative AI, a form of AI that generates new content by employing a combination of learning approaches, has become prominent in research and is influencing various sectors, including healthcare.

ML, as one of the major domains of AI, is an approach that enables systems to learn from data and improve their performance over time as new data becomes available to the system. In the realm of numerical data, ML models can be used for prediction, classification, and clustering.^12^ Prediction algorithms such as regression can be employed to recommend personalized treatment options, forecast price, size and other numerical data, while classification algorithms can be used to categorize data into different groups such as disease type or health outcomes. For example, ML models can enable systems to predict shortages and manage the supply chain effectively. This feature empowers health systems to allocate scarce resources more efficiently through smart management. In processing perceptual data, ML can play a key role in recognizing and analyzing images and video, voice, and text. An example of image recognition is analyzing medical images like X-rays to assist radiologists in diagnosing various diseases and health conditions.^8^ Video recognition, for example, can help with patient monitoring, allowing for function and behavior tracking, accident detection, and quick alerts to medical staff in hospitals or long-term care facilities and so on. Voice recognition, another noteworthy application, is utilized to transcribe medical dictations and its advancement can potentially reduce the risk of errors in patient records. Text recognition can be beneficial for analyzing EMR, such as prescription information, and identifying potential drug interactions.

ML models are categorized based on their fundamental approaches, resulting in three primary families: supervised, unsupervised, and reinforcement learning. Supervised learning is an approach that relies on labelled data to predict the labels. This type of model is used when prior information about the expected output values exists. Predictions (regression) and classification models can be examples of supervised learning techniques that require labelled data for training algorithms. In contrast, unsupervised models, such as clustering, extract patterns from unlabelled data by grouping similar unlabelled data without the need for supervision. Reinforcement learning is a sophisticated model that a machine learns from its own experience. The machine trains itself by using feedback from and interacting with its surroundings. This approach is particularly effective in scenarios with well-defined rules and outcomes, like robotic-assisted surgery.^4,12,19–22^

NLP, another significant domain within AI, enables computers to understand, analyze, and generate human language. The interactions between humans and computers can occur through both text and speech, allowing models to be utilized for knowledge extraction, summarization, translation, and other applications. Large language models (LLMs), a subset of generative AI, such as ChatGPT and many others, are specialized applications within the broader field of NLP. They leverage deep neural networks, an important subset of ML algorithms, to understand and generate human language. These models are initially trained on big text data gathered from a wide variety of sources. Depending on the requirements the model is further refined for specific tasks to adapt the model's understanding and provide a more suitable output to those tasks. To date, some LLMs have been trained on numerous data sources from DH solutions, such as EMR.^4,22^

Regulatory and HTA bodies need to evaluate the unique characteristics of LLMs. They should ensure the developers of LLM-embedded DHTs transparently outline the strategies to mitigate bias in their models. Additionally, they should disclose their methods to manage the generation of misleading information and to address data security and patient privacy concerns. New assessment methods are required to evaluate the accuracy, intended and unintended effects on patients’ outcomes, continuous monitoring, rigorous testing of DH solutions, and the return on investment for healthcare systems.^23,24^ Additionally, regulatory and HTA agencies can compile a list of requirements related to certain characteristics for LLMs used in DHTs. These requirements include but are not limited to, clinical validation studies, potential risk-benefit analysis, privacy safeguards, security measures, performance metrics, usability criteria, interoperability standards, and post-market surveillance. Additionally, robust frameworks and guidelines must address LLM-specific considerations such as transparency about model workings, training data, and ethical standards. The intersection of DH solutions, AI, and RWE may end in personalizing therapeutic options and health care to optimize efficiency and enhance the sustainability of health systems.^25^

## Conclusion

The intersection of DH and AI represents a transformative frontier in healthcare, promising to improve patient outcomes, streamline healthcare delivery, and optimize decision-making processes. A clearer understanding of DH and AI concepts, along with their accurate usage, expedites the development of high-quality standards and frameworks, resulting in the generation of regulatory and HTA-grade evidence and outcomes. An initial step is to ensure accurate and distinct usage of DH and AI terminologies and avoid the interchangeability of the two. This categorization facilitates the development of standard frameworks and category-specific guidelines, which helps to establish quality standards, develop validation methods, and define comparable endpoints for measuring the impact of DH solutions. The health systems need to ensure DHTs solutions follow certain requirements and evaluate their performance in the real world. It is also important to prioritize DHT groups that are more used for collecting RWD and delivering direct health benefits to patients. In the healthcare sector, relying on high-quality evidence is crucial for making informed decisions with greater certainty. High-quality evidence requires well-designed DHTs, which require developers to adhere to established guidelines and assessment criteria. Therefore, regulatory and HTA bodies must develop clear frameworks and category-specific guidelines to select the highest-quality technologies that improve patient outcomes and decision-making certainty. Enhancing our understanding and familiarity with AI and DH concepts contributes to achieving these objectives and promotes a collaborative approach that involves healthcare providers, clinical experts, and patients, as well as AI developers, and regulatory and reimbursement authorities. As DH and AI continue to shape the future of healthcare, this collaboration is essential for advancing the ethical implementation of DH and AI solutions that align with healthcare standards and improve patient outcomes throughout the inevitable transformation of the health system.
